# Stable water isotopes reveal the onset of bud dormancy in temperate trees, whereas water content is a better proxy for dormancy release

**DOI:** 10.1093/treephys/tpae028

**Published:** 2024-02-28

**Authors:** Manuel G Walde, Bénédicte Wenden, Isabelle Chuine, Arthur Gessler, Matthias Saurer, Yann Vitasse

**Affiliations:** Ecosystem Ecology, Forest Dynamics, Swiss Federal Institute for Forest, Snow and Landscape Research WSL, Zürcherstrasse 111, 8903 Birmensdorf, Switzerland; INRAE, Univ. Bordeaux, UMR Biologie du Fruit et Pathologie, 71 av. Edouard Bourlaux, 33140 Villenave d'Ornon, France; CEFE, Univ. Montpellier, CNRS, EPHE, IRD, 1919 route de Mende, 34293 Montpellier, France; Ecosystem Ecology, Forest Dynamics, Swiss Federal Institute for Forest, Snow and Landscape Research WSL, Zürcherstrasse 111, 8903 Birmensdorf, Switzerland; Institute of Terrestrial Ecosystems, ETH Zurich (Swiss Federal Institute of Technology), Universitätsstrasse 16, 8092 Zurich, Switzerland; Ecosystem Ecology, Forest Dynamics, Swiss Federal Institute for Forest, Snow and Landscape Research WSL, Zürcherstrasse 111, 8903 Birmensdorf, Switzerland; Ecosystem Ecology, Forest Dynamics, Swiss Federal Institute for Forest, Snow and Landscape Research WSL, Zürcherstrasse 111, 8903 Birmensdorf, Switzerland

**Keywords:** chilling, climate chamber experiments, forcing, stable isotope labelling, tree phenology

## Abstract

Earlier spring growth onset in temperate forests is a visible effect of global warming that alters global water and carbon cycling. Consequently, it becomes crucial to accurately predict the future spring phenological shifts in vegetation under different climate warming scenarios. However, current phenological models suffer from a lack of physiological insights of tree dormancy and are rarely experimentally validated. Here, we sampled twig cuttings of five deciduous tree species at two climatically different locations (270 and 750 m a.s.l., ~ 2.3 °C difference) throughout the winter of 2019–20. Twig budburst success, thermal time to budburst, bud water content and short-term ^2^H-labelled water uptake into buds were quantified to link bud dormancy status with vascular water transport efficacy, with the objective of establishing connections between the dormancy status of buds and their effectiveness in vascular water transport. We found large differences in the dormancy status between species throughout the entire investigation period, likely reflecting species-specific environmental requirements to initiate and release dormancy, whereas only small differences in the dormancy status were found between the two studied sites. We found strong ^2^H-labelled water uptake into buds during leaf senescence, followed by a sharp decrease, which we ascribed to the initiation of endodormancy. However, surprisingly, we did not find a progressive increase in ^2^H-labelled water uptake into buds as winter advanced. Nonetheless, all examined tree species exhibited a consistent relationship between bud water content and dormancy status. Our results suggest that short-term ^2^H-labelled water uptake may not be a robust indicator of dormancy release, yet it holds promise as a method for tracking the induction of dormancy in deciduous trees. By contrast, bud water content emerges as a cost-effective and more reliable indicator of dormancy release.

## Introduction

Temperate trees exert a significant influence on global water and carbon cycles and provide important feedback to the earth’s climate system (Richardson et al. 2013). Throughout their evolutionary history, they have optimized the timing of their spring growth resumption to local climate to avoid late-spring frost damages while maximizing the competitiveness for resources (Chuine 2010, Lenz et al. 2016, Baumgarten et al. 2023). Temperate tree species have therefore developed adaptive strategies to adjust their spring phenology based on abiotic requirements such as chilling (cold temperature during winter dormancy), forcing (temperature promoting cell growth after dormancy release) and photoperiod (reviewed in Delpierre et al. 2016).

Thus, different models have been proposed to predict the spring phenology of deciduous and evergreen trees over the last 50 years which include these main environmental factors (Cannell 1989, Hänninen 1990, Chuine 2000). Controlled experiments have been developed in recent years to characterize the different phases of dormancy along with associated environmental cues (e.g., Laube et al. 2014, Flynn and Wolkovich 2018, Baumgarten et al. 2021, Walde et al. 2022) but have been rarely used to improve phenological models. Yet, insights into physiological mechanisms that occur during winter bud dormancy progression should provide the basis for predicting phenology under an unprecedentedly rapid changing climate (Basler 2016). Therefore, a growing interest in the molecular, physiological and structural regulations of bud dormancy has recently emerged, especially thanks to technological advances and thorough investigations in fruit trees planted in a warmer climate (Liu and Sherif 2019, Fadón et al. 2020, Pan et al. 2021, Yang et al. 2021). However, the comprehension of the main metabolic and inter- and intracellular molecular signalling components that lead to the physiological changes involved in the progression of winter bud dormancy is still incomplete (Velappan et al. 2017, Considine and Foyer 2023). Lang et al. (1987) distinguished three consecutive stages of dormancy, called paradormancy (regulated by physiological factors outside the affected organ), endodormancy (regulated by physiological factors inside the affected organ) and ecodormancy (regulated by environmental factors). These three stages are convenient to describe the phenological status of the buds through winter dormancy even though growing evidence demonstrates that they are not totally separated in time, but gradually overlap (Cooke et al. 2012, Fadón et al. 2020). Investigating the transition from endodormancy to ecodormancy, i.e., when bud meristems start to get sensitive to warmer temperatures, would improve the accuracy of model predictions under warmer climatic scenarios (Chuine et al. 2016).

Major structural and physiological changes during dormancy initiation were found in a couple of perennial plants (reviewed in Savage and Chuine 2021). First, under abscisic acid (ABA) regulation, a build-up of callose plugs at cells’ plasmodesmata takes place in autumn, isolating living cells from each other, both in the buds and in the phloem cells (Tylewicz et al. 2018). Those plugs form a so-called dormancy sphincter complex that prevents water flow from the stem tissue into the buds and drastically reduce the metabolic and physiological activities (reviewed in Rinne and van der Schoot 2003). This symplastic isolation is restored during winter (supposedly after a certain exposure to chilling conditions) due to a shift in the metabolic balance from net callose synthesis to net callose degradation (Rinne et al. 2001). It is not clear, however, when this restoration of symplastic pathway between cells occurs during winter and which environmental cues are driving it. It is also unknown if this restoration is responsible for dormancy release or a prerequisite for other processes, ultimately resulting in bud swelling and budburst (Fouché et al. 2023). Additionally, plugs of tannin-like molecules build-up in the xylem at the junction between the bud and the stem at the same period have been observed in some species, isolating the buds from the main vascular system.

Recent work suggests that the dormancy release of deciduous trees is also tightly coupled to the concentration of ABA and carbohydrates (reviewed in Fadón et al. 2020). Sweet cherry tree buds, for instance, typically increase ABA concentration during endodormancy before decreasing it again during ecodormancy, whereas sucrose concentrations steadily increase during endodormancy until maximum concentrations are reached at the beginning of ecodormancy (Vimont et al. 2021, Chmielewski and Götz 2022, Götz and Chmielewski 2023). In addition, the activation of inter- and intracell communications through aquaporins seem to play an important role in spring for cells turgescence and bud swelling in deciduous trees (Yooyongwech et al. 2008). Bud water content of sweet cherry and peach decreased during endodormancy and increased again during ecodormancy before budburst (Yooyongwech et al. 2009, Götz et al. 2014, Kaufmann and Blanke 2017).

Here, we quantified the bud water content and applied an isotopic labelling technique to track changes in water transport from shoot to bud through the vascular system from autumn to spring, and we assessed the link between bud water content and short-term water uptake into buds and dormancy initiation and release. Twig cuttings of five deciduous tree species from two sites in northern Switzerland were regularly sampled from October 2019 to April 2020 and were incubated at 20 °C either in deionized water until budburst or in ^2^H (deuterium)-labelled water for 24 h. The twigs incubated in deionized water were used to determine dormancy status (dormancy depth evaluated as the amount of days at forcing conditions required to budburst), whereas the ones incubated in ^2^H-labelled water were used to determine the bud water content and to track the short-term water uptake into the buds over the incubation period. This novel experiment addressed the following questions:

(i) How do the investigated tree species differ in the progression of dormancy within and between the two climatically different sites?
(ii) How does bud water content change during dormancy progression and to which extent is it correlated with dormancy depth?
(iii) Could stable water isotope labelling of twig cuttings be used to quantify the dormancy status of deciduous tree species?

We expected a large difference in dormancy progression between and among different species due to their species-specific temperature and photoperiod requirements. We expected warmer temperatures at the low-elevation site to delay the timing of leaf coloration and the onset of endodormancy. Further, we expected a gradual increase in short-term water uptake with increasing chilling when exposed to 20 °C.

## Materials and methods

### Site description and sampling

**Figure 1:**
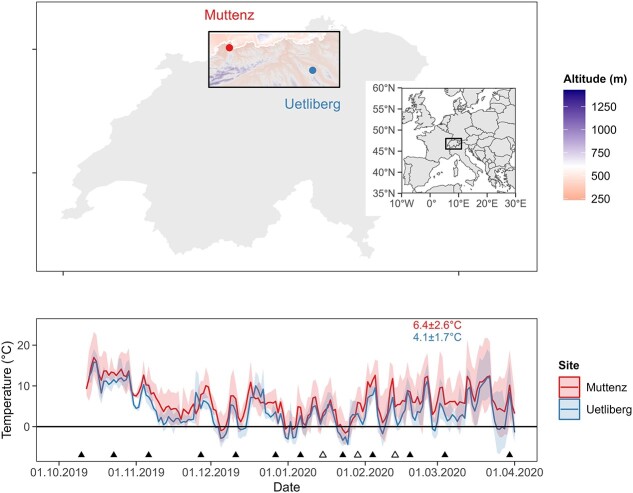
Location of the sampling sites and air temperature at the sampling sites. Samples were collected at 270 m a.s.l. (Muttenz) and between 700 and 800 m a.s.l. (Uetliberg). The lines in the lower panel represent daily mean air temperature, and shaded areas show daily minimum and maximum temperatures. Average air temperatures ± average daily standard deviation of air temperature throughout the examined period are indicated in corresponding colours. Sampling campaigns are indicated with filled triangles (all parameters) and empty triangles (bud water content and short-term water uptake only).

Twig cuttings were sampled from two mixed forests in northern Switzerland with an altitudinal difference of ~500 m and substantially different climatic conditions. The low-elevation site is located in Muttenz near Basel at 270 m a.s.l. (47°32′27.6″N 7°38′49.2″E) and the high-elevation site is located on Uetliberg near Zurich between 700 and 800 m a.s.l. (47°21′15.1″N 8°29′16.0″E). The average air temperature was recorded every hour, 2 m above ground, using temperature loggers installed at tree trunks at the north-exposed side without any direct sun exposition (HOBO MX2203). The average temperature and the daily average standard deviation of the temperature from 11 October 2019 to 1 April 2020 was 6.4 ± 2.6 °C for the site Muttenz and 4.1 ± 1.7 °C for the site Uetliberg (Figure 1).

At each site, twigs were collected on five species: *Acer pseudoplatanus* L., *Carpinus betulus* L., *Fagus sylvatica* L., *Quercus petraea* Liebl. and *Tilia cordata* Mill. For clarity and brevity, hereafter, we refer to each species by its English genus name, i.e., maple, hornbeam, beech, oak and lime, respectively. We chose this set of species due to their contrasting sensitivity to temperature and photoperiod during dormancy (see Walde et al. 2022).

We selected five healthy adult trees for each species and site (>10 m height, > 30 years old) at the first sampling date and repeatedly harvested two 60-cm twig cuttings from each tree using a 7-m long pole pruner during 12 sampling campaigns from October 2019 to April 2020, i.e., approximately every 2 weeks (Table S1 available as Supplementary data at *Tree Physiology* Online). Twigs were put into plastic bags directly after sampling and were carried to the research institute within a few hours. At the research institute, all residual leaves were removed, twigs were recut at the bottom and the cut surfaces were placed in transparent plastic boxes filled with tap water on the same day to keep the lowermost 5 cm of the twig underwater, and they were then stored at 2 °C over night. In addition to the 12 sampling campaigns, 3 additional campaigns were conducted during January and February 2020 to obtain a higher resolution of short-term water uptake into buds, as we expect the transition from endodormancy to ecodormancy to occur during this period.

### Monitoring of budburst

**Figure 2:**
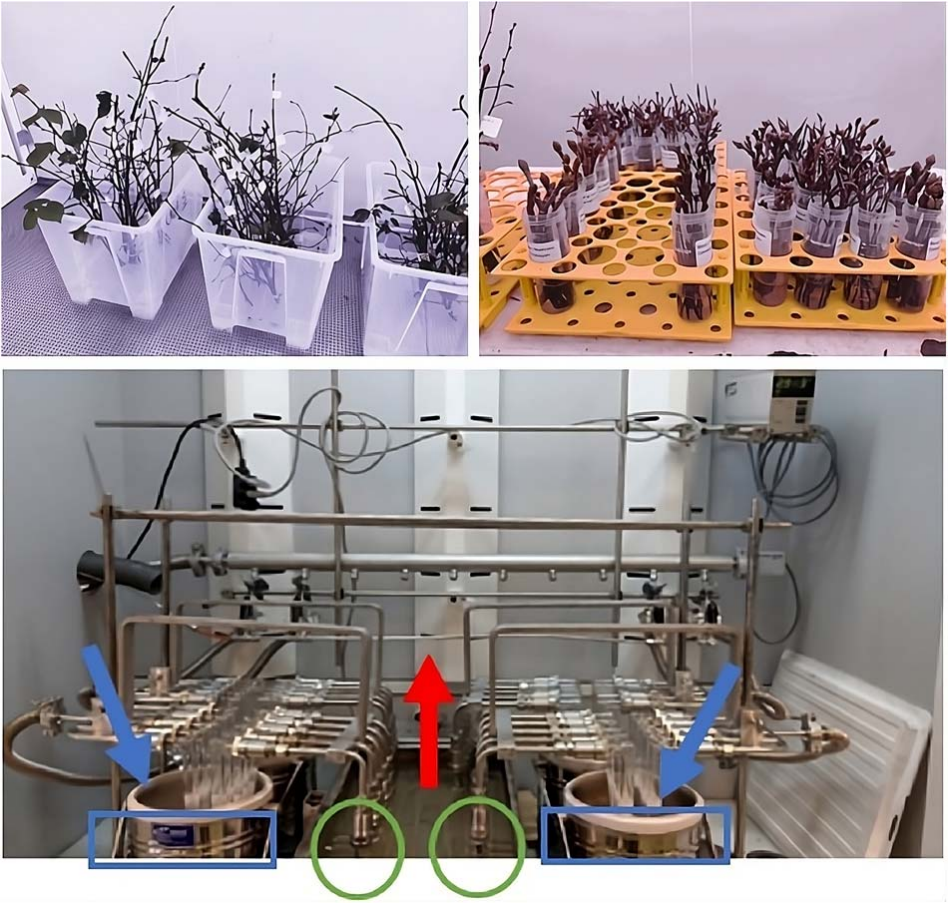
Graphical illustration of the setting. Illustrated are twigs from one sampling campaign placed in transparent plastic boxed for the determination of dormancy depth and budburst success (top left), the labelling of twig cuttings (top right) and water extraction by the self-made CVD (bottom). The CVD uses low pressure and elevated temperature to evaporate water vapour (red arrow) from the samples (green circles) that condensates later in a cold trap (blue arrow) that uses liquid nitrogen as freezing agent (blue rectangles).

The following morning after the sampling, the first twig of each individual was pruned to ~ 50 cm and was placed into transparent plastic boxes such that the lowest most 5 cm of the twig was submerged by deionized water (see Figure 2, top left). These boxes were exposed to 20 °C forcing temperature and 24-h photoperiod in a climate chamber to evaluate the dormancy status. A cutting length of ~ 50 cm was chosen to include a representative number of buds for each twig and to be able to frequently recut the base of the twig (by c. 0.5 cm) until budburst to prevent vessel occlusion. Twigs were recut and the deionized water was replaced at weekly intervals until budburst. Spring phenology (i.e., leaf-out) of cuttings was monitored twice a week using a five-stage categorical scale (Vitasse 2013). This classification consists of no visible development (stage 0); bud swelling (stage 1); budburst and partially visible leaves (stage 2); fully emerged but folded, crinkled or pendant leaves (stage 3) and fully unfold leaves (stage 4, see Figure S1 available as Supplementary data at *Tree Physiology* Online). Once the earliest bud per twig reached a new stage, the day of year was recorded. The success rate of budburst was assessed 2 weeks after the earliest bud reached stage 4 by counting the number of buds that reached at least stage 2 compared with the total number of buds (Baumgarten et al. 2021). The thermal time required to budburst (stage 2) after exposure to forcing conditions (20 °C) was used to determine the dormancy depth under natural conditions. We focused on the budburst stage since budburst is the earliest clearly visible indicator of leaf emergence for all species, whereas the accomplishment of later stages may depend on the availability of resources that could be limited when using twig cuttings (Vitasse and Basler 2014). The climate chamber provided stable temperatures within ±1 °C of the target value and was equipped with halogen lamps (Philips MASTER TL-D) with a photosynthetic photon flux density of 50 μmol m^2^ s^−1^ at bud height.

### Monitoring of short-term water uptake using ^2^H-labelled water

The second twig of each individual was pruned to ~ 10-cm long pieces, each containing one single bud at the uppermost part. Then, those pieces were placed into 30-mL plastic tubes that were previously filled with 10 mL of deuterium-labelled water (δ^2^H ~ 2000‰) and were exposed to the same forcing conditions (see Figure 2, top right). We used a small cutting length of ~ 10 cm to reduce the distance between the water gage and the uppermost bud in order to speed up the uptake of labelled water into the buds. After 24 h, the uppermost bud of each piece of twig was abscised just above the base of the bud and the samples were stored at −20 °C until water extraction. The samples were weighed before and after water extraction to determine the bud water content.

Bud water was extracted using a self-made cryogenic vacuum distillation (CVD) based on the model presented in West et al. (2006). A CVD system extracts water enclosed in a sample by evaporating it in one compartment and then condensing it again in another compartment (Figure 2, bottom). The main components of the CVD system utilized are a water bath maintained at 80 °C in the first compartment and u-tubes submerged in liquid nitrogen in the second compartment (see Orlowski et al. 2013, Diao et al. 2022). The entire process was accelerated with a vacuum pump (BS2212, Brook Crompton Ltd, Doncaster, UK) that kept the pressure below 0.05 mbar during the extraction. The extraction was carried out for 2 h to extract all tissue water. After the extraction, the vacuum pump was stopped and dry nitrogen gas was pumped into the CVD system. Then, the u-tubes were taken out of the liquid nitrogen and were detached from the CVD system. The u-tubes were sealed with rubber plugs, and the extracts were thawed at room temperature before transferring them into glass vials (350 μL or 2 mL, depending on the extracted water amount; Infochroma AG, Goldau, Switzerland) using a pipette. Water stable isotopes of the extracts, δ^2^H and δ^18^O, were measured with a high temperature conversion elemental analyser coupled to a Delta^Plus^ XP isotope ratio mass spectrometer (TC/EA-IRMS; Finnigan MAT, Bremen, Germany) with a precision of 1‰ for δ^2^H and 0.2‰ for δ^18^O.

**Figure 3:**
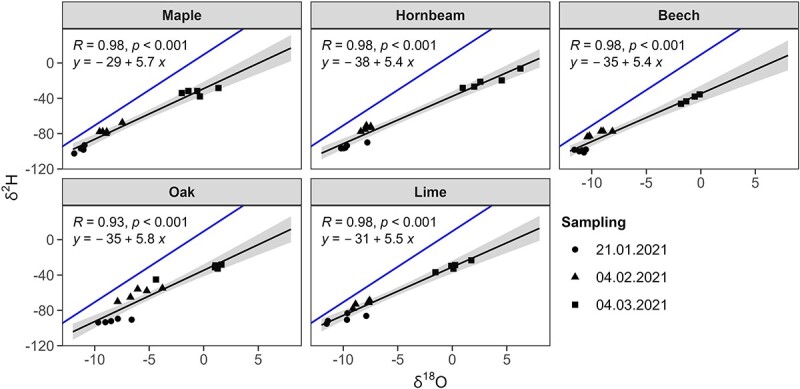
Relationship between natural abundance oxygen (δ^18^O) and hydrogen isotope signatures (δ^2^H) of bud water. This species-specific linear relationship between oxygen and hydrogen isotopes was used to retrieve the excess of label taken-up into buds (short-term water uptake). The blue line indicates the global annual average relationship between oxygen and hydrogen isotopes (global meteoric water line).

The δ^2^H and δ^18^O inside the buds can vary during the winter season due to evaporation from the buds, whereas the relationship between the two isotopes should remain stable. To determine the relationship between the natural isotope abundance of δ^2^H and δ^18^O during the course of winter, we performed three additional sampling campaigns during winter 2020–21 (Figure 3).

This determined relationship was used in a next step to predict the expected (natural abundance) background δ^2^H in each sample (background δ^2^H) of the ^2^H-labelling study at each sampling date based on the measured δ^18^O and the species-specific linear relationship between δ^18^O and δ^2^H with intercept β_0_ and slope β_1_ (Eq. (1)).


(1) \begin{equation*} {\mathrm{\delta}}^2{\mathrm{H}}_{\mathrm{background}}={\mathrm{\beta}}_0+{\mathrm{\beta}}_1\cdot{\mathrm{\delta}}^{18}{\mathrm{O}}_{\mathrm{sample}} \end{equation*}

Then, deuterium (^2^H) atom% equivalent to the background δ^2^H, sample δ^2^H and label δ^2^H were calculated using Eq. (2) with *R*_standard_ being the isotopic composition of Vienna Standard Mean Ocean Water.


(2) \begin{equation*} \mathrm{atom}\%=\frac{100\cdot{R}_{\mathrm{standard}}\cdot \left(\frac{\mathrm{\delta}^2\mathrm{H}}{1000}+1\right)}{1+{R}_{\mathrm{standard}}\cdot \left(\frac{\mathrm{\delta}^2\mathrm{H}}{1000}+1\right)} \end{equation*}

The ^2^H atom% of samples, background and label were used to calculate the short-term uptake of labelled water (%), i.e., the excess ^2^H over background ^2^H was calculated using Eq. (3). Short-term uptake of labelled water (%) reflects therefore the excess of deuterium that was able to pass from the labelled water in the tubes to the buds via the vascular system of the twig after 24 h at 20 °C.


(3) \begin{align*} &\mathrm{Uptake}\ \mathrm{of}\ \mathrm{labelled}\ \mathrm{water}\ \left(\%\right)=\nonumber\\ &\qquad\frac{\mathrm{atom}{\%}_{\mathrm{sample}}-\mathrm{atom}{\%}_{\mathrm{background}}}{\mathrm{atom}{\%}_{\mathrm{label}}-\mathrm{atom}{\%}_{\mathrm{background}}}\cdot 100 \end{align*}

On 24 January 2020, we also analysed the short-term water uptake in twig tissues below the base of the buds (c. 0.5 cm) in order to demonstrate whether water flow in the twigs was possible, but it was blocked prior to reaching bud meristems. The same species-specific equation already used to calculate the short-term water uptake into bud was used to calculate the water uptake into twig tissue next to the bud since we found the slope of the linear relationship between twig tissue δ^18^O and δ^2^H to be similar to the slope of bud tissue (Figure S2 available as Supplementary data at *Tree Physiology* Online).

### Data analysis and statistics

Budburst success, thermal time to budburst, bud water content and the short-term uptake of labelled water were modelled using generalized linear mixed effect models. Budburst success and dormancy depth were modelled with a binomial regression and a polynomial regression, respectively, with sampling dates (continuous variable), species (categorical variable with five levels) and sites (categorical variable with two levels) and every possible two-way interaction as fixed effects and donor tree identity as random effect. In the latter model, the dependent variable thermal time to budburst model was transformed by the natural logarithm and the sampling dates were parametrized by a second-order polynomial to normalize the dormancy depth data and to accurately model the species-specific non-linear progression of dormancy depth during winter. We included only sampling campaigns after the first frost (i.e., 27 November 2019) for the assessment of dormancy depth as the tree species showed inconsistent non-linear thermal time to budburst patterns before the first frost happened.

Bud water content and short-term water uptake were modelled similarly, but using sampling dates as categorical variables (15 levels), to better model nonlinear outcomes and to be able to compare individual sampling campaigns. Species-specific Pearson correlation coefficients were calculated between budburst success, dormancy depth, bud water content and short-term water uptake. All correlations were tested without sampling campaigns 1–3 (i.e., 10 October 2019–7 November 2019), because for these correlations, our aim was to focus on the physiological state of the bud related to internal dormancy, while during these campaigns, leaves were still present and were potentially photosynthetically active, which could influence the bud dormancy through interactions with sugars and ABA (Chao et al. 2007).

All analyses and plots were performed in R v.4.1.1 (R Core Team 2023). All models were fitted using the R package glmmTMB (v1.1.5) that handles a wide range of statistical distributions as well as model extensions such as zero-inflation, heteroscedasticity and autocorrelation (see https://github.com/glmmTMB/glmmTMB). All figures were generated using ggplot2 (v3.4.0). The package car (v3.1-1) was used to run ANOVAs and package emmeans (v1.8.4-1) was used to run post hoc tests using Tukey’s honestly significant difference to test for significant differences between species, sampling dates and sites. A type II ANOVA has been used to evaluate budburst success (*n* = 583), whereas type III ANOVAs have been used to evaluate dormancy depth (*n* = 442), water content (*n* = 620) and short-term water uptake (*n* = 620).

## Results

### Species-specific dormancy progression at the different sites

**Figure 4:**
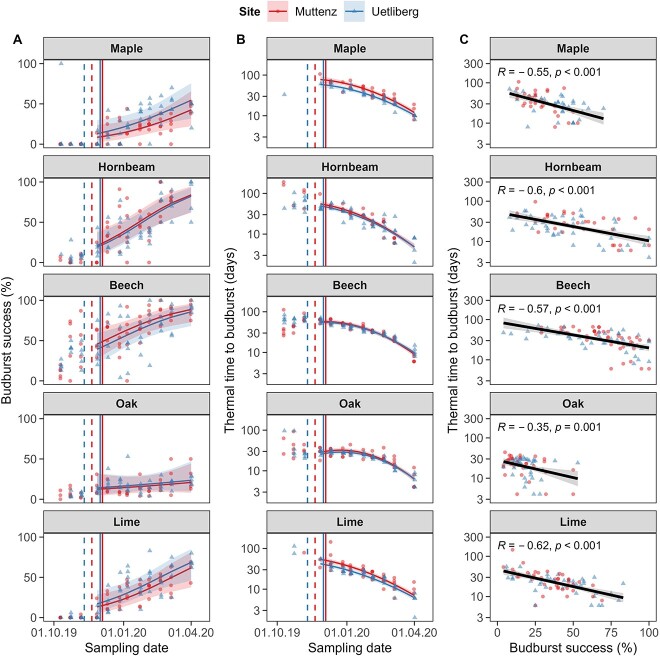
(A) Fraction of budburst success, (B) thermal time to budburst and (C) correlation between budburst success and thermal time to budburst at 20 °C and constant photoperiod forcing condition. Solid lines and shaded areas represent model predictions and corresponding 0.90 confidence intervals, respectively, whereas points represent the underlying raw data. The different sites are illustrated by triangles for Uetliberg (high-elevation site) and circles for Muttenz (low-elevation site). The first day with a minimum temperature <0 °C is indicated with dashed lines. The first day with average temperatures <0 °C is indicated with solid lines.

Overall, most (>85%) beech and oak cuttings from both sites and hornbeam cuttings from Uetliberg were able to budburst throughout the entire experimental period, whereas maple and lime cutting as well as hornbeam cuttings from Muttenz showed a considerably lower budburst ability particularly during autumn and early winter (Figure S2 available as Supplementary data at *Tree Physiology* Online). All species increased budburst success with the progression of winter with significant differences among species (Figure 4A). Oak, for instance, showed the smallest increase in budburst success from 15% at the first sampling date to 45% at the last sampling date, whereas lime showed the highest increase from 0% at the first to 80% at the last. Most of the maple and lime cuttings did not budburst before the first frost event happened, whereas most beech cuttings were able to budburst throughout the entire examined period. Budburst success did not differ between sites for any species investigated (Table 1 and Figure 4A).

**Table 1:** Impact of the sampling date, species, site and their interactions on budburst success and thermal time to budburst at 20 °C and 24-h photoperiod. Table shows chi square (χ^2^), degree of freedom (*df*) and *P*-value. Significant results (*P* < 0.05) are indicated in bold.

	**Budburst success**			**Thermal time to budburst**		
Fixed effect	χ^2^	*df*	*P*-value	χ^2^	*df*	*P*-value
(Intercept)	NA	NA	NA	2897.6	1	**<0.001**
Sampling date	57	1	**<0.001**	366.4	2	**<0.001**
Species	60.5	4	**<0.001**	65.9	4	**<0.001**
Site	0.2	1	0.651	6.7	1	**0.010**
Sampling date:species	6.2	4	0.182	88.3	8	**<0.001**
Sampling date:site	0.1	1	0.723	3.0	2	0.220
Species:site	2	4	0.741	4.6	4	0.327

Thermal time to budburst differed between sampling date, species, site and the interaction between sampling date and species (Figure 4B and Table 1). Overall, maple and beech required significantly more thermal time to budburst than hornbeam, oak and lime (all *P*s < 0.001). In addition, hornbeam showed a faster decrease in thermal time to budburst than the other species (*P* < 0.001). Maple and lime cuttings from the low-elevation site, Muttenz, required significantly more thermal time to budburst than cuttings from the high-elevation site, Uetliberg. All species showed a strong negative linear relationship between the budburst success and thermal time to budburst (all *P*s < 0.001, Figure 4C).

### Bud water content and bud water uptake during dormancy

**Figure 5:**
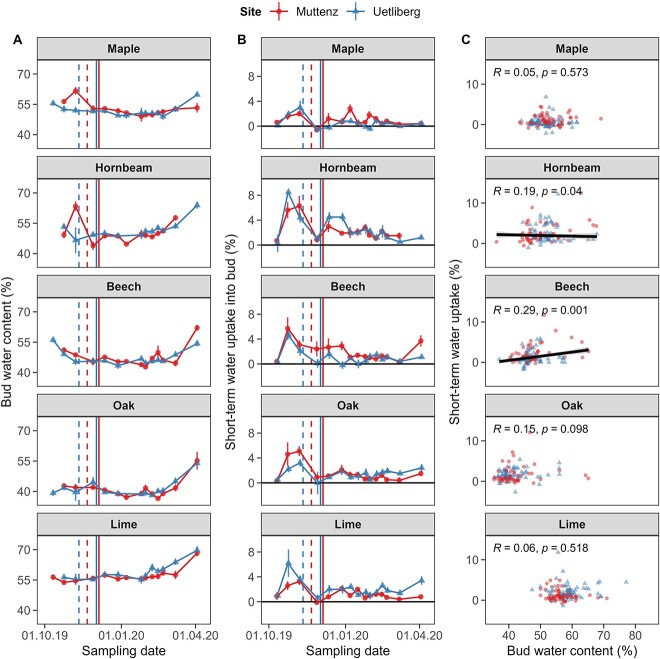
(A) Effect of winter progression on bud water content, (B) short-term water uptake into buds and (C) correlation between bud water content and short-term water uptake after 24 h at 20 °C and constant photoperiod forcing condition. Opaque points and error bars represent mean and corresponding standard errors, respectively, while solid lines and shaded areas in (C) represent model predictions and corresponding 0.90 confidence intervals and transparent points represent the underlying raw data. The different sites are illustrated by triangles for Uetliberg (high-elevation site) and circles for Muttenz (low-elevation site). The first day with minimum temperature <0 °C is indicated with dashed lines. The first day with average temperatures <0 °C is indicated with solid lines.

Bud water content depended significantly on the sampling date, species, their interaction and the interaction between sampling date and site (Table 2 and Figure 5A). For instance, the bud water content significantly declined for beech from the first sampling date (10 October 2019, leaves predominantly green) to the fourth sampling date (27 November 2019, i.e., after first frost and finished leaf senescence), whereas the water content of oak did not change during this period (*P* = 0.002 and *P* = 0.989, respectively). From the second to the third sampling date (i.e., 23 October 2019–6 November 2019), maple and hornbeam individuals from the lower elevation site (Muttenz) showed a striking increase in bud water content that was significantly higher compared with trees sampled at the higher elevation site (Uetliberg, both *P*s < 0.001). Overall from December to February, the bud water content was found to be the highest for lime with ~ 57 ± 3% (mean ± standard deviation) and the lowest for oak with ~ 40 ± 4% (Figure 5A). All species showed a significant increase in bud water content from the penultimate to the last sampling date before budburst even though some species required >2 weeks from the last sampling date to budburst (all *P*s < 0.001; see Table S2 available as Supplementary data at *Tree Physiology* Online).

**Table 2:** Impact of the sampling date, species, site and their interactions on bud water content and short-term water uptake after 24 h exposure to 20 °C and constant light. Table shows chi square (χ^2^), degree of freedom (*df*) and *P*-value. Significant results (*P* < 0.050) are indicated in bold.

	**Bud water content**			**Short-term water uptake**		
Fixed effect	χ^2^	*df*	*P*-value	χ^2^	*df*	*P*-value
(Intercept)	292.4	1	**<0.001**	1.0	1	0.313
Sampling date	87.7	13	**<0.001**	45.5	13	**<0.001**
Species	53.6	4	**<0.001**	1.7	4	0.790
Site	0.3	1	0.600	1.2	1	0.282
Sampling date:species	188.1	52	**<0.001**	127.5	52	**<0.001**
Sampling date:site	53.2	13	**<0.001**	14.2	13	0.360
Species:site	5.7	4	0.222	30.1	4	**<0.001**

Short-term water uptake into buds depended significantly on the sampling date, the interaction between sampling date and species and the interaction between site and species (Table 2). At the beginning of our investigation (i.e., 10 October 2019), no short-term water uptake into buds was observed (Figure 5B). Then, during the progression of leaf senescence (i.e., 23 October and 6 November 2019), large amounts of short-term water allocation were detected. From 6 to 27 November, all species, except beech from Muttenz, decreased water uptake from relatively high values to almost zero (all *P*s < 0.001), likely marking a physiological barrier blocking the passage of water occurring at the interface between the twig and the bud. From late November to the penultimate sampling date, the short-term water uptake was minimal but was not null for all species. During this period, hornbeam showed often significantly higher short-term water uptake than most other species (all *P*s < 0.050). Beech cuttings from Muttenz and lime cuttings from Uetliberg showed a significant increase in short-term water uptake from the penultimate to the last sampling campaign before budburst, thereby showing patterns similar to the bud water content (all *P*s < 0.001).

Short-term water uptake increased for beech (*P* = 0.001) and decreased for hornbeam (*P* = 0.040) with an increasing bud water content, while no relationship between short-term water uptake and bud water content was found for any other species (Figure 5C). Consequently, the bud water content did not provide reliable information about how vessel conductivity changed during winter progression.

**Figure 6:**
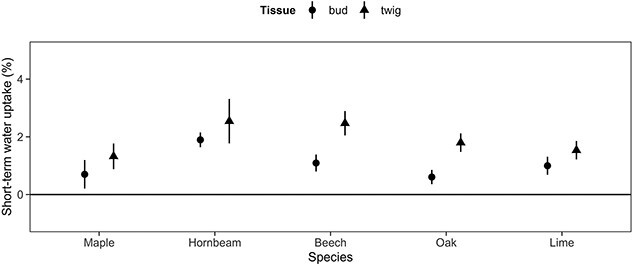
Species-specific short-term water uptake into different tissues after 24 h at forcing condition (20 °C and 24-h photoperiod) at sampling campaign 9 (i.e., 24 January 2020). Opaque points and error bars represent mean and corresponding standard errors.

At the ninth sampling date (i.e., 24 January 2020), which took place at the coldest day of the winter 2019–20, the short-term water uptake into twigs underneath the buds was investigated and all species tended to incorporate more labelled water into twigs than into buds with a significant difference for beech (*P* = 0.012) and oak (*P* = 0.024, see Figure 6). This result indicates that, at this time, a large portion of labelled water remained locked below the base of the buds.

### Short-term water uptake and water content in relation to dormancy status

**Figure 7:**
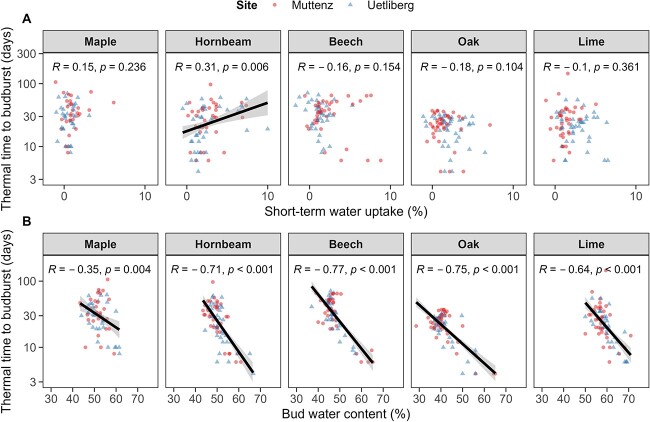
(A) Correlation between thermal time to budburst and short-term water uptake and (B) bud water content after dormancy induction (i.e., after 07 November 2019). Solid lines and shaded areas in represent model predictions and corresponding 0.90 confidence intervals and points represent the underlying raw data. The different sites are illustrated by triangles for Uetliberg (high-elevation site) and circles for Muttenz (low-elevation site).

No correlation was found between thermal time to budburst and short-term water uptake in all species except for hornbeam, where a decrease in short-term water uptake was associated with decreasing thermal time to budburst (Figure 7A). Lime, but no other species, showed a significant increase in budburst success with increasing short-term water uptake (Figure S4A available as Supplementary data at *Tree Physiology* Online). However, all species showed a strong negative correlation between thermal time to budburst and bud water content (Figure 7B), and all species, except maple, showed a strong positive correlation between budburst success and bud water content (Figure S4B available as Supplementary data at *Tree Physiology* Online).

## Discussion

Our experimental study shows that, for all species, budburst success increased and thermal time to budburst decreased during the progression of winter and that those two variables are strongly correlated, whereas intraspecific differences between the sampling sites were either small or not existent for these two parameters. We found strong isotopic signals in autumn for all species, which indicate that the short-term water uptake into buds is high during leaf colouration before decreasing to a minimum after completion of leaf senescence, i.e., once leaves have fallen, likely marking the onset of endodormancy. Surprisingly, no gradual increase in the short-term water uptake into buds was detected as winter progressed, but an abrupt increase occurred shortly before budburst. However, we found a gradual increase in the bud water content as winter progressed, which correlated strongly with the thermal time requirement to budburst for all investigated species. This suggests that bud water content appears to be a reliable and simple marker for dormancy depth during ecodormancy unlike the short-term water uptake using water isotopes.

### Species-specific dormancy progression

Similar to previous work, all investigated temperate trees species increased budburst success and decreased thermal time to budburst as winter progressed (e.g., Baumgarten et al. 2021). However, we found significant differences between species, which reflect their different sensitivities to environmental cues. For example, beech and oak were able to budburst throughout the entire investigation period, whereas the other three species only successfully budburst after being exposed to a minimum period of chilling. This outcome is consistent with previous work that showed that some temperate species require a minimum amount of chilling to budburst (Laube et al. 2014, Baumgarten et al. 2021). Budburst success rate did not significantly differ between individuals of the same species growing at different sites. However, thermal time to budburst of maple, hornbeam and lime was lower throughout the entire investigated period for cuttings from the higher elevation site Uetliberg, likely reflecting a higher exposure to chilling temperatures in this site. Thus, increasing winter temperatures may slow down the advance of spring phenology in some tree species in temperate ecosystems due to decreasing or delaying chilling conditions (Fu et al. 2015, Wang et al. 2020, Beil et al. 2021, Zhang et al. 2022). However, this effect could also be caused by intraspecific differences arising from genetic adaptation to site-specific environmental conditions (Vitasse et al. 2013).

### Short-term water uptake as a proxy for dormancy status

Remarkably, the investigation of bud water under natural conditions revealed that the slope between oxygen (δ^18^O) and hydrogen isotopes (δ^2^H) is less pronounced than expected from the relationship commonly found in the global meteoric water line (Figure 3). The stronger accumulation of the heavier oxygen isotope (^18^O) isotope compared with the heavier hydrogen isotope (^2^H) in the buds indicates Rayleigh fractionation (see Gonfiantini 1986). This suggests that buds are losing some water by evaporation through the cuticle during winter dormancy (Wiegand 1906), which is supported by a shift in δ^18^O from 21 January 2021 to 4 March 2021 by up to 10‰ (Figure 3). However, the bud water content of all investigated species did not significantly change from the beginning of December to the end of February, as found in Essiamah and Eschrich (1986). This suggests that the evaporated water was continuously replaced from the adjacent tissue to protect the buds from desiccation.

Using water isotopes, we detected large fluctuations in the short-term water uptake in autumn during the progression of leaf senescence for all species. All species showed the highest short-term water uptake during the second (late October) or third (early November) sampling campaign, while the lowest values were found during the first (mid-October) and fourth sampling campaigns (end of November). This pattern seems related to the progress of leaf senescence, which was completed (all leaves were either coloured or fallen) between the first and fourth sampling campaigns for all investigated individuals. During this period, the photosynthetic apparatus is broken down through catabolic enzymes, and valuable nutrients such as nitrogen and phosphorus are re-allocated from the leaf to storage tissues before abscission (Matile 2000, Keskitalo et al. 2005, Fracheboud et al. 2009). Our results indicate that during this process, water transport in buds was possible likely through the vascular system and was probably necessary for the nutrient and sugar resorption. We attributed the sharp decline in short-term water uptake thereafter for all species to the onset of endodormancy, a period characterized by low plasmodesmal and low passive water transport between cells due to high callose deposition and strong repression of aquaporin genes, respectively (Rinne and van der Schoot 2003, Yooyongwech et al. 2008, Fouché et al. 2023). However, further investigations that combine genetic and physiological approaches would be necessary to examine the coupling of endodormancy induction and leaf senescence (e.g., Yooyongwech et al. 2009, 2015, Fouché et al. 2023). New experiments oriented towards this goal would therefore help to improve the accuracy of existing phenological models.

Surprisingly, no gradual increase in short-term water uptake was observed during ecodormancy for any species, and we did not find evidence for increasing water flow from the twig into the bud with increasing chilling exposure, which was contrary to previous work (e.g., Aloni and Peterson 1997, Rinne et al. 2001) but was in line with a recent study conducted on sweet cherry (Fouché et al. 2023). Thus, vascular water transport into buds appears to be non-functional from the end of leaf senescence until at least just before budburst for the investigated species either due to obstruction of water flow or immature xylem (Goodwin 1967, Xie et al. 2018). Our results therefore indicate that the increase of bud water content in late winter and early spring may take place through the apoplastic and/or symplastic pathways from water stored in adjacent tissues, which seems sufficient to prevent bud desiccation during dormancy, but it is probably insufficient to supply the increasing water demand during bud burst and leaf-out (Savage and Chuine 2021). However, it remains unclear at which stage of spring phenology the vascular pathway is restored. Therefore, the origin of water sources involved in cell enlargement (bud swelling) within buds before budburst and leaf unfolding is still unclear and deserves more attention in future experiments. Since our ^2^H-labelling approach requires long-distance transport from the cut along the twig to the bud, a potential way to track short-distance apoplastic and or symplastic water transport could be the injection of labelled water with microsyringes into the twig at short distances from the terminal bud.

### Bud water content as a proxy for dormancy status

Overall, the bud water content decreased after leaf senescence and started to increase for all species during early spring, c. 6–8 weeks before budburst, likely corresponding with the period where buds start to respond to warm temperatures (i.e., ecodormancy). This finding makes the quantification of bud water content potentially interesting as a low-tech method to investigate the dormancy status of temperate deciduous tree species during spring. The increase in dry and fresh weights of flower primordia with winter progression has been used as a marker of dormancy break in fruit trees (e.g., Brown and Kotob 1957, Legave and Garcia 1982). Basler and Körner (2014) found increasing bud areas for deciduous forest trees with progression of spring under natural conditions, but to the best of our knowledge, no study has examined the bud water content change as winter progresses for forest trees leaf buds.

We found linear relationships between the bud water content and thermal time to budburst for all species after dormancy induction. Therefore, tracking the evolution of bud water content during winter could be a more reliable proxy for the progress of winter dormancy in deciduous forest trees than short-term vascular water uptake capacity. Magnetic resonance imaging on fruit trees revealed that most water is bound within cells at the initiation of dormancy, and is gradually freed under forcing conditions, while at the same time, substantial enlarging of flower primordia occurs long before signs of budbreak become visible (reviewed in Faust et al. 1997). Therefore, our observation provides further evidence for the tight linkage between dormancy depth and cold hardiness, where the increasing water content with progression might be attributed to deacclimation (Vitra et al. 2017, North and Kovaleski 2023). Yet, there might be no consistent pattern between bud water content and cold hardiness between species as more freezing resistant species, such as maple and hornbeam, had, on average, higher bud water contents during peak dormancy depth and during dormancy release than more susceptible species such as beech and oak (Lenz et al. 2013, Vitra et al. 2017).

The strong correlation between budburst success and thermal time to budburst for all species illustrates that both variables are to some extent interchangeable and important metrics of the physiological dormancy status of temperate deciduous tree species (Baumgarten et al. 2021). This finding is indicating obstructions at the initiation of dormancy that strongly reduced water flow for some buds (high thermal time to budburst) or even prevented it completely for others (low budburst success), which were then gradually degraded with progress of dormancy. We suppose decreasing ABA concentrations to be a key driver of this transformation, as high ABA concentrations have been shown to increase plasmodesmal callose deposition and to downregulate aquaporin action (Tylewicz et al. 2018, Pan et al. 2021, Fouché et al. 2023).

Interestingly, twig cuttings of maple and hornbeam showed significantly lower bud water content at the high-elevation site, Uetliberg, compared with cuttings from the low-elevation site, Muttenz, at the third sampling campaign (beginning of November). This sampling campaign was the first one with daily average temperatures <5 °C at Uetliberg and took place only 4 days before the occurrence of the first frost. Therefore, endodormancy of maple and hornbeam at Uetliberg might have been induced earlier in response to a rapid decrease of temperature, leading to a reduction in bud water content to ensure the protection of their buds from potential frost damages (Vitra et al. 2017).

## Conclusions

Overall, our study highlighted large differences in budburst success and thermal time to budburst between common European species, likely reflecting species-specific chilling requirements and sensitivities to forcing temperature, whereas much smaller differences were found between the two study sites. All species showed a strong short-term water uptake during leaf senescence, likely reflecting efficient water transport through the vascular system for nutrient resorption. Once the leaves were fully coloured or had fallen off, an abrupt decline of short-term water uptake was detected, marking the onset of endodormancy. However, in contrast to our hypothesis, we did not find evidence for an increasing water uptake through the vascular system as a marker for endormancy release, which was associated with decreasing thermal time to budburst. Buds of temperate deciduous trees are unlikely to be taking up water through the vascular system during endo- and ecodormancy. Therefore, our methodology is only partially suitable as a tracer of dormancy status of temperate deciduous trees, namely for tracking endodormancy onset. Nevertheless, this water isotopic labelling may reveal new insights if applied in a higher temporal resolution during leaf coloration and leaf fall to precisely track the initiation of endodormancy, and further experiments would be needed to quantify which climatic conditions induce dormancy onset besides plant internal factors. Interestingly, bud water content seems to be a reliable and simple proxy for thermal time to budburst during ecodormancy for all investigated species in contrast to short-term water uptake using water isotopes.

## Supplementary Material

SI_Dormancy_Track2_tpae028

## Data Availability

The data used in this manuscript will be archived with Dryad Digital Repository: https://doi.org/10.5061/dryad.sn02v6x99.
