# Supplementary material for: Stable water isotopes reveal the onset of bud dormancy in temperate trees, whereas water content is a better proxy for dormancy release
Source: Tree Physiol. 2024 Feb 28;44(4):tpae028. doi: 10.1093/treephys/tpae028 (PMC11016847; doi:10.1093/treephys/tpae028)
Supplement: SI_Dormancy_Track2_tpae028 [file si_dormancy_track2_tpae028.docx]

# Appendix


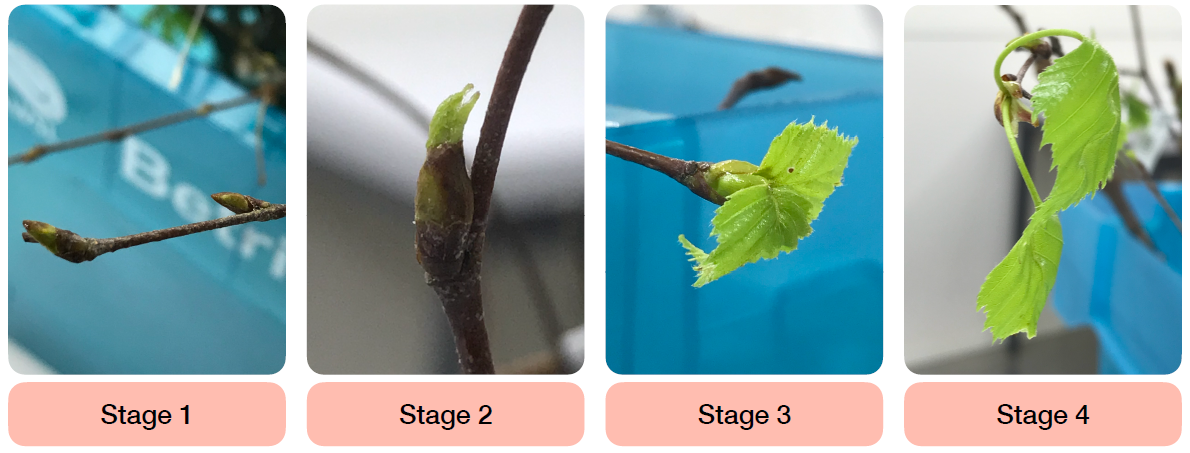

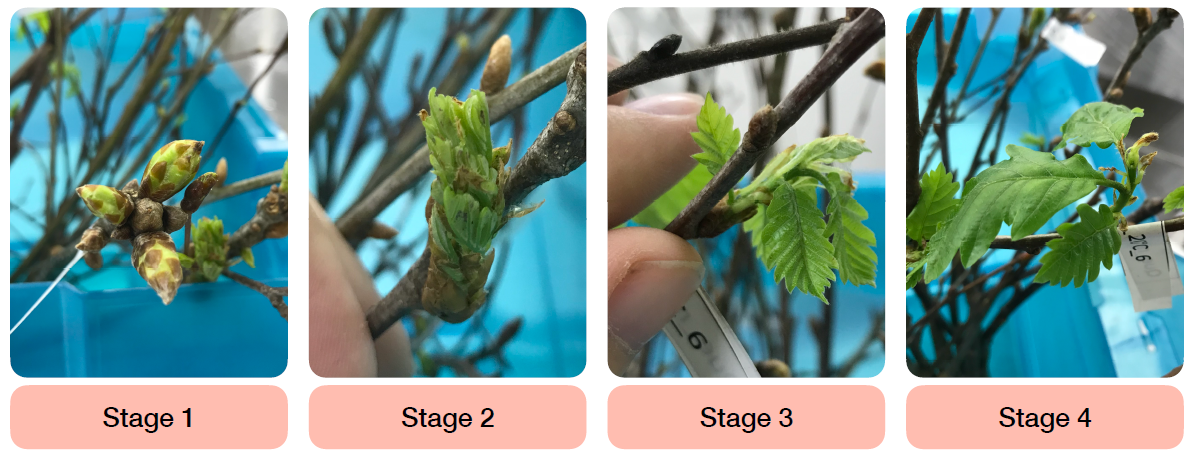


**Fig. S1.** A visual representation of the spring phenological stages used to classify leaf-out of deciduous tree species. Illustrated are representative examples for oak (top) and birch cuttings (bottom).


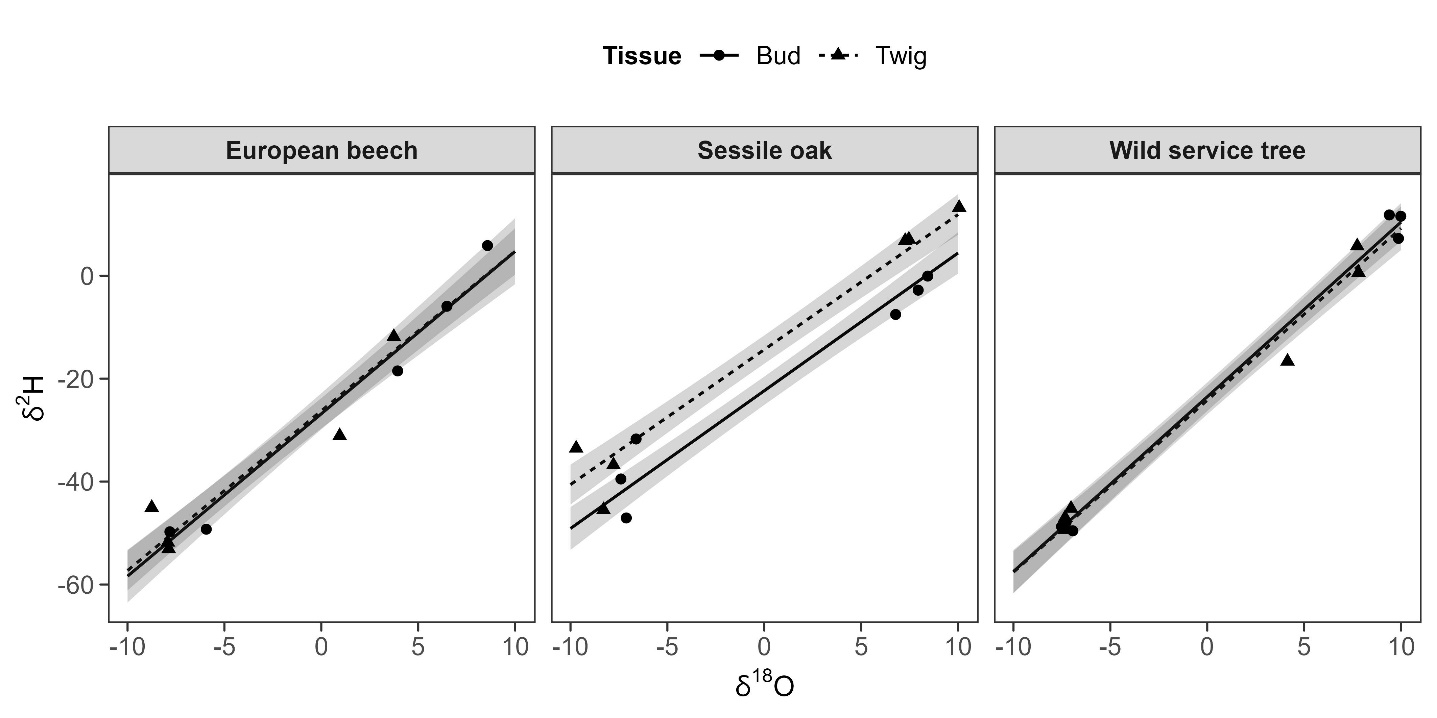


**Fig. S2.** Relationship between oxygen (δ^18^O) and hydrogen isotopes (δ^2^H) contained in water within buds and twigs of potted tree saplings measured during winter 2022.


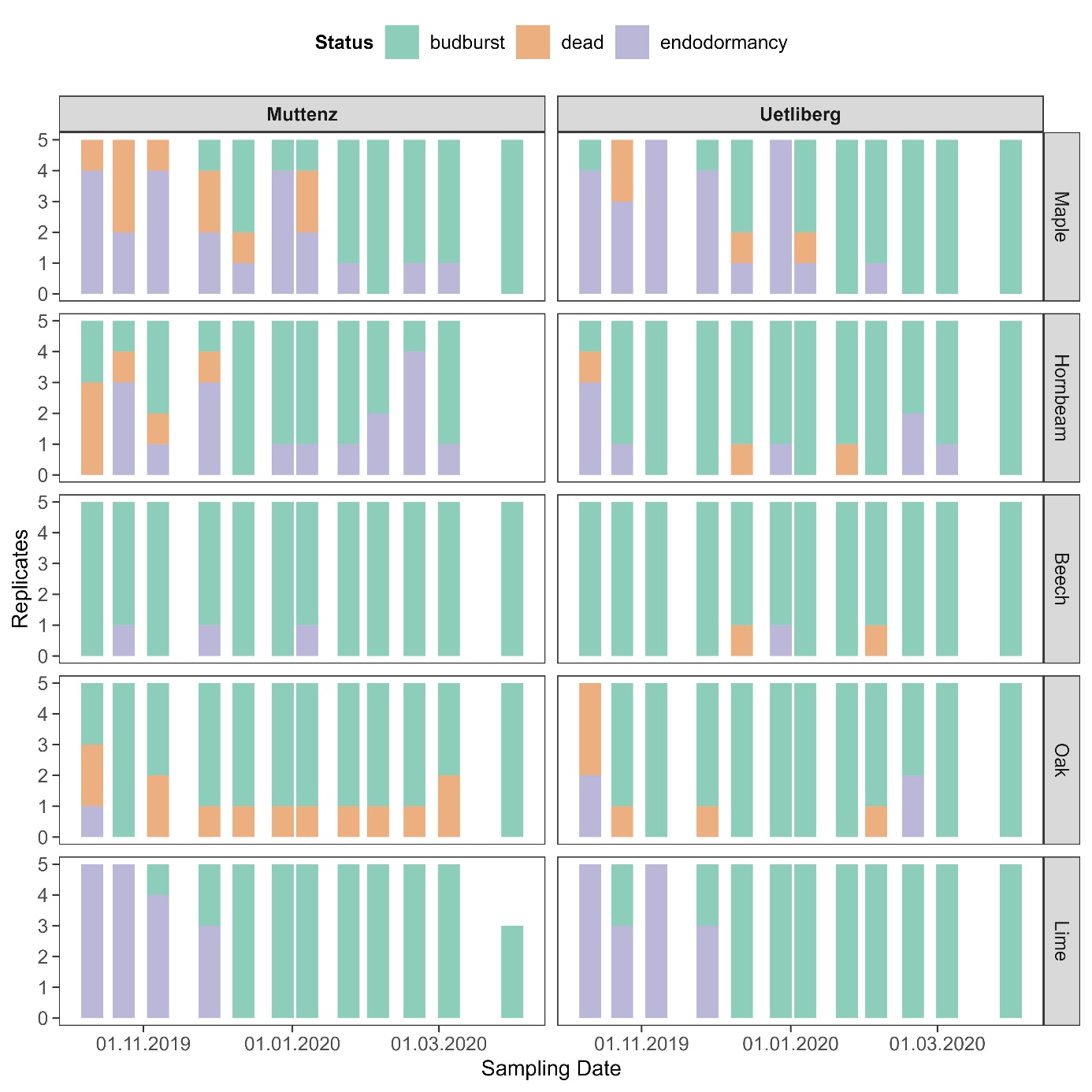


**Fig. S3.** Status of the cuttings at the end of the experiment dependent on sampling date. Green (budburst) indicates cuttings that budburst before the end of April, orange (dead) indicates cuttings that died in the climate chamber, and lilac (endodormancy) indicates cuttings that survived until the end of the experiment but were not able to budburst even after several months of 20°C and 24 h photoperiod forcing conditions.


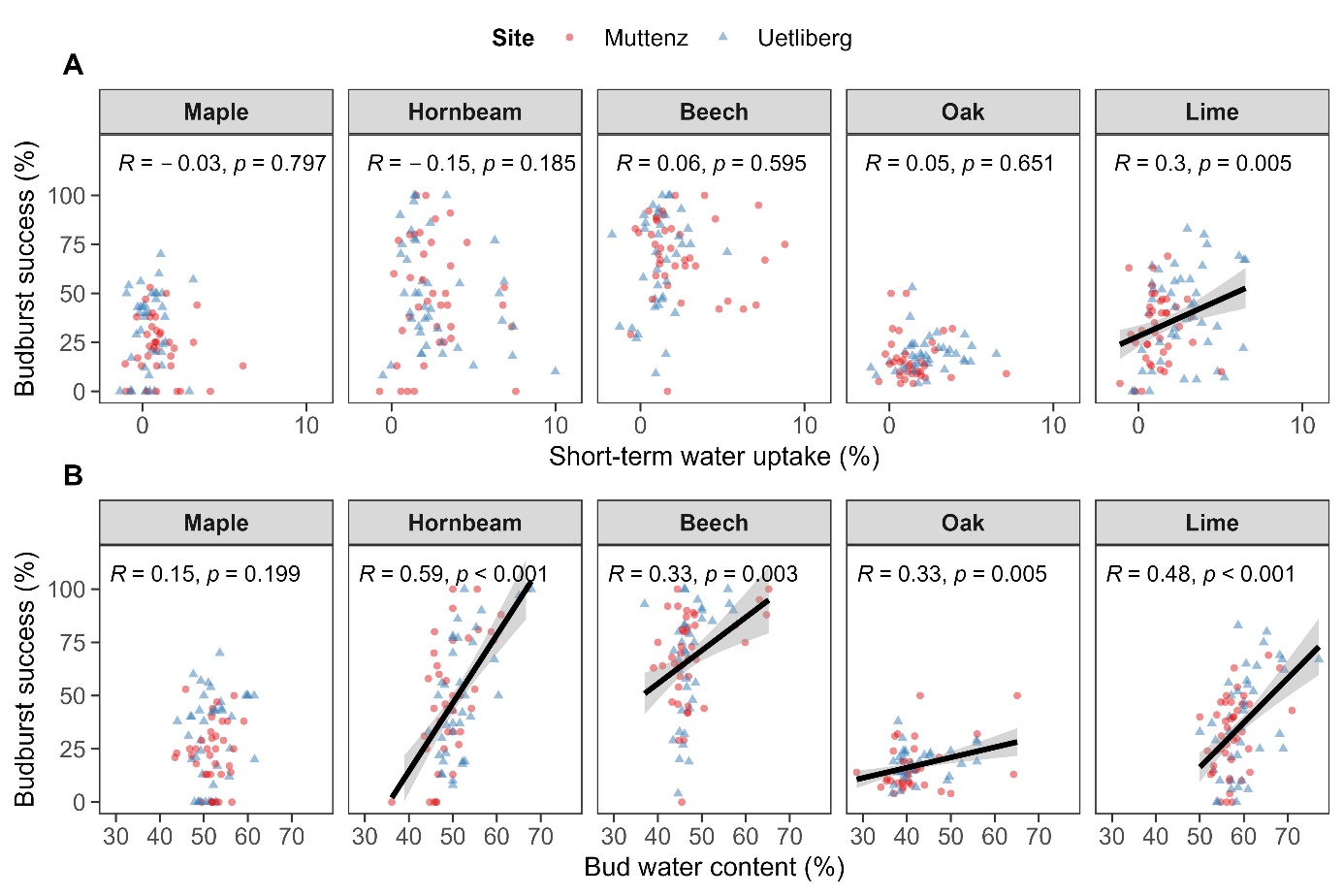


**Fig. S4.** (A) Correlation between budburst success rate and short-term water uptake and (B) bud water content. Solid lines and shaded areas in represent model predictions and corresponding 0.90 confidence intervals and points represent the underlying raw data. The different sites are illustrated by blue triangles for Uetliberg (high elevation site) and red circles for Muttenz (low elevation site).

**Table S1.** Overview of sampling campaigns represented as date and day of the year (DoY).

| **Campaign** | **Date** | **DoY** | **Comment** |
| --- | --- | --- | --- |
| 1 | 10.10.2019 | 283 |  |
| 2 | 23.10.2019 | 296 |  |
| 3 | 06.11.2019 | 310 |  |
| 4 | 27.11.2019 | 331 |  |
| 5 | 11.12.2019 | 345 |  |
| 6 | 27.12.2019 | 361 |  |
| 7 | 06.01.2020 | 6 |  |
| 8 | 15.01.2020 | 15 | additional sampling for isotope analysis |
| 9 | 24.01.2020 | 24 |  |
| 10 | 29.01.2020 | 29 | additional sampling for isotope analysis |
| 11 | 06.02.2020 | 37 |  |
| 12 | 13.02.2020 | 44 | additional sampling for isotope analysis |
| 13 | 19.02.2020 | 50 |  |
| 14 | 05.03.2020 | 65 |  |
| 15 | 31.03.2020 | 91 |  |

**Table S2.** Predicted day of the year (DoY) of budburst. DoY of budburst was reconstructed using the thermal time to budburst observed in the climate chamber and the observed temperature at the site. For all species the sampling campaign before budburst was used for the prediction.

|  |  | **Predicted DoY of budburst** | | |
| --- | --- | --- | --- | --- |
| **Site** | **Species** | **Average** | **Earliest** | **Latest** |
| Muttenz | Maple | 112 | 105 | 115 |
| Muttenz | Hornbeam | 81 | 79 | 82 |
| Muttenz | Beech | 104 | 103 | 107 |
| Muttenz | Oak | 103 | 100 | 111 |
| Muttenz | Lime | 104 | 91 | 115 |
| Uetliberg | Maple | 108 | 106 | 109 |
| Uetliberg | Hornbeam | 103 | 97 | 106 |
| Uetliberg | Beech | 110 | 106 | 114 |
| Uetliberg | Oak | 103 | 100 | 108 |
| Uetliberg | Lime | 102 | 97 | 106 |
